# 3D Pharmacophoric Similarity improves Multi Adverse Drug Event Identification in Pharmacovigilance

**DOI:** 10.1038/srep08809

**Published:** 2015-03-06

**Authors:** Santiago Vilar, Nicholas P. Tatonetti, George Hripcsak

**Affiliations:** 1 Department of Biomedical Informatics, Columbia University Medical Center, New York, NY, USA

## Abstract

Adverse drugs events (ADEs) detection constitutes a considerable concern in patient safety and public health care. For this reason, it is important to develop methods that improve ADE signal detection in pharmacovigilance databases. Our objective is to apply 3D pharmacophoric similarity models to enhance ADE recognition in Offsides, a pharmacovigilance resource with drug-ADE associations extracted from the FDA Adverse Event Reporting System (FAERS). We developed a multi-ADE predictor implementing 3D drug similarity based on a pharmacophoric approach, with an ADE reference standard extracted from the SIDER database. The results showed that the application of our 3D multi-type ADE predictor to the pharmacovigilance data in Offsides improved ADE identification and generated enriched sets of drug-ADE signals. The global ROC curve for the Offsides ADE candidates ranked with the 3D similarity score showed an area of 0.7. The 3D predictor also allows the identification of the most similar drug that causes the ADE under study, which could provide hypotheses about mechanisms of action and ADE etiology. Our method is useful in drug development, screening potential adverse effects in experimental drugs, and in drug safety, applicable to the evaluation of ADE signals selected through pharmacovigilance data mining.

Post-marketing unexpected adverse drugs events (ADEs) cause an important damage in patient health with the consequent risk for patients and high care expenses^1^,^2^. For this reason, an early detection of the ADEs is an urgent objective to improve the functionality of the health-care system. Different regulatory actions can be carried out once unexpected ADEs are detected that go from warnings in drug labels to the drug withdrawal from the market. Moreover, ADEs are an important cause for the high degree of attrition in experimental drugs and an early detection can help to save efforts in the drug development process^3^.

Different types of computational approaches using protein target and pathway data have been published to discover associations between drugs and ADEs or possible mechanisms of action for the adverse reactions^4^,^5^. Cheminformatic ligand-based approaches exploiting the idea that similar molecules have similar properties have also been applied in the assessment of adverse drug reactions^6^. Computer-aided methods are very useful to predict toxicological effects and select candidates in the drug development process without undesirable properties. As an example, multiple quantitative structure activity/property relationship (QSAR/QSPR) models have been established to predict a variety of toxicological properties^7^, such as carcinogenicity^8^ or reproductive and developmental toxicity^9^, as well as different types of absorption, distribution, metabolism and excretion (ADME) properties, i.e. aqueous solubility, plasma protein binding or blood-brain barrier penetration^10^,^11^. However, the prediction of complex clinical adverse events offers important limitations due to the variety of possible mechanism of actions responsible for the occurrence of the ADE in a patient^7^. Despite this fact, *in silico* QSAR/QSPR models have been developed from preclinical and clinical data to improve the early prediction of different complex ADEs, such as QT prolongation, phospholipidosis or hepatotoxicity^12^. Some studies integrated adverse drug reactions data with cheminformatic fingerprint-based modeling through the use of Laplacian-modified Bayesian models, nearest neighbor, support vector machines or correlation analysis, among others^13^,^14^,^15^. The models showed applicability in preclinical safety pharmacology and prediction of adverse drug reactions.

Moreover, applicability of these methods goes beyond experimental drugs and they have also shown potential as safety tools applied to drugs already in the market through the signal detection enhancement in pharmacovigilance studies^16^,^17^. In reference to this topic, different pharmacovigilance methodologies have been applied to study and discover clinical ADEs caused by drugs in the market. The majority of these pharmacovigilance methods extract signals or drug-ADE associations generated through data mining of health-care databases, such as the FDA Adverse Event Reporting System (FAERS)^18^, Electronic Health Records (EHRs) or claims data^19^,^20^,^21^,^22^. Although the analysis of pharmacovigilance data offers great potential and good results in the identification of adverse effects^19^,^20^,^21^,^22^,^23^, the existence of confounding factors reduce the efficiency of this type of methods^20^. As it was shown previously, the existent methods still provide a high false positives candidates rate that makes difficult the signal interpretation. However, alternative analysis of the selected drug-ADE signals provided by cheminformatic approaches can improve the signal detection generating sets of enriched drug-ADE candidates. In previous studies, we achieved a significant signal enhancement in FAERS and EHRs through the use of molecular fingerprint-based modeling in the study of complex ADEs, such as rhabdomyolysis and pancreatitis^16^,^17^. Other types of similarities, such as 3D pharmacophoric similarity, adverse event profile similarity and target profile similarity also yielded models with applications in pharmacovigilance signal detection^24^.

In this study we generated a large scale multi-type drug-ADE predictor integrating 3D molecular structure similarity extracted through a pharmacophoric approach with a reference standard of multiple drug-ADEs associations extracted from SIDER database^25^. The multi-type drug-ADE predictor can be applicable under different scenarios: 1) ADE screening of candidates as experimental drugs in the development process, and 2) with important implications in the pharmacovigilance of drugs already existent in the market through the re-evaluation of drug safety signals extracted from pharmacovigilance data mining studies. Moreover, the nature of the multi-ADE predictor allows the researcher to identify for each candidate the most similar drug in the reference standard along with the available ADE information. This fact can lead in some cases to the establishment of hypothesis for the drug candidates about possible mechanism of action related to the ADEs.

Our results showed that the implementation at large scale of 3D cheminformatic models in drug safety data is helpful in signal detection and facilitates the decision making to further study some of the selected candidates. Figure 1 illustrates the flowchart followed in the development of the current study.

## Methods

### 3D pharmacophoric similarity data (matrix Ma)

*Drug structure preparation:* We downloaded the drug structures from DrugBank^26^. Proteins and large peptides were not included in the calculation due to the complexity to determine the most stable 3D molecular structures. Drugs were mapped to the SIDER database (our reference standard) establishing 853 drugs by which pharmacophoric data was calculated. DrugBank provided the bioactive conformations with specified chiral centers for some drugs. When no chirality information is available, we generated a maximum of three enantiomers for each drug using the module LigPrep from the Schrödinger package [Schrödinger, version 9.2, LLC, New York, USA, 2011]. We also generated different drug protonation states depending on the pH = 7.0. Geometry of the different structures was initially optimized.

*Conformational analysis:* We performed a conformational analysis for all the drugs using the module Macromodel from Schrödinger. The calculation was performed in water as an implicit solvent to diminish possible intra-molecular forces and generate more extended conformations more in accordance with biologically active structures. Non-bonded cut-off distances were extended to 4.0, 8.0 and 20.0 Å for H-bond, *van der Waals* and electrostatic forces respectively. The selected conformational search engine was Monte Carlo Multiple Minimum (MCMM). For simplicity, only the global minimum energy structure according to the OPLS_2005 force field was retained for the next step.

*Shape screening and similarity searching:* shape screening calculations were performed using the Phase module from Schrödinger package [Schrödinger, version 9.2, LLC, New York, USA, 2011]. In the calculation we used the 3D molecular structures determined through the previous conformational analysis as templates. Each drug conformation under evaluation was aligned to each 3D template identifying similar shape and pharmacophoric features between each pair of drugs. A 3D similarity score (Phase Sim property) between each pair of drugs was calculated using as a volume scoring the pharmacophoric type. The structures are treated as sets of pharmacophore sites with a radius of 2 Å. The 3D score measures the overlap volume between pharmacophoric sites of the same type present in both drugs and ranges from 0 (maximum dissimilarity) to 1 (maximum similarity). The calculated 3D score between the structures A and B is defined as:
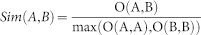
where O(A,B) is the pharmacophoric overlap between the structure A and structure B and max(O(A,A),O(B,B)) is the maximum of the self-overlaps.

*Construction of 3D similarity matrix (matrix Ma):* We integrated all 3D similarity scores between pairs of drugs (Phase Sim property) in an 853×853 drug-drug similarity matrix called Ma. In this matrix, rows and columns heads represent drugs and each cell includes the similarity between drugs. Values in the diagonal representing similarity of each drug against itself are set 0; through this step we will generate a leave-one-out procedure in the posterior development of the 3D predictor.

### ADE reference standard (matrix Mb)

SIDER^25^ resource was used as the initial reference standard for drug-ADE associations. SIDER is a database with 4,192 side effects related to 996 drugs with 99,423 drug-ADE associations (prevalence = 0.024). Side effect information is extracted from package inserts, drug labels and public documents. Although more studies would be necessary to confirm some adverse reactions described in SIDER, this database is a valuable source of relationships between drugs and ADEs. The data was transformed into a drug-ADE binary matrix with the columns representing adverse reactions and the rows the drugs included in the study. Each cell includes the value 1 if the drug-ADE is present in SIDER or 0 if the drug-ADE is not described in our initial reference standard. After mapping drugs between SIDER and DrugBank and considering only ADEs with at least five drugs, our final matrix Mb contains 853 rows (drugs) and 1,780 columns (ADEs). The number of drug-ADE associations (value 1 in the matrix) is 87,151 and the prevalence is 0.057.

### Data integration and multi-ADE model development: generation of matrix Mc

**Figure 2:**
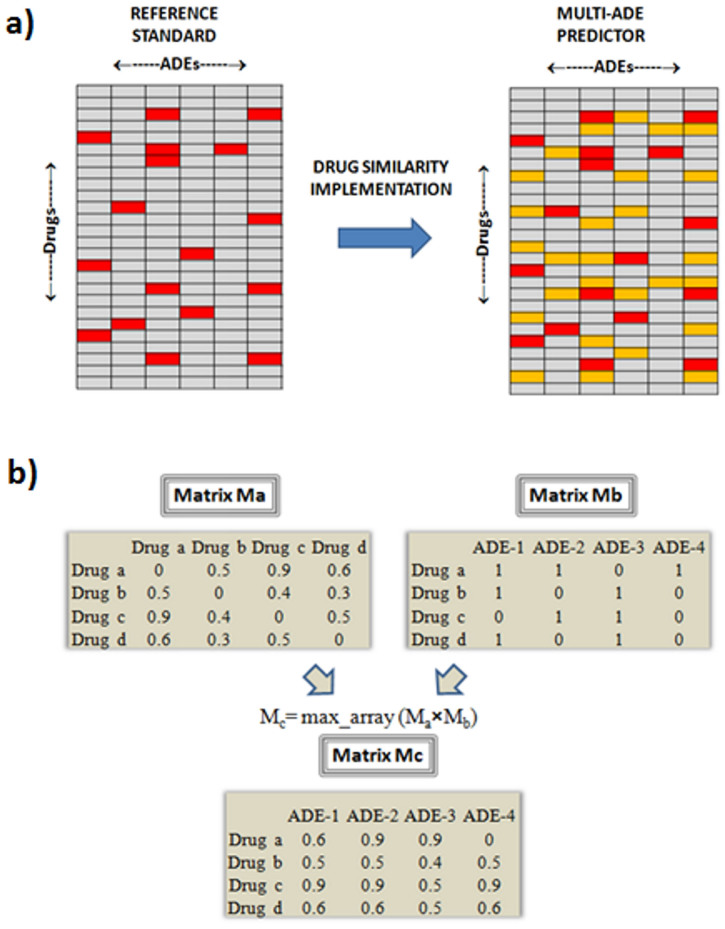
Panel a) In the drug×ADE matrix (reference standard) is implemented the 3D drug similarity to develop the multi-ADE predictor. Drugs with associated ADEs in the reference standard are colored in red. Drugs predicted to be associated with the ADEs are colored in orange (new candidates); Panel b) Integration of the data in the model development. In the product of the drug-drug 3D similarity matrix (Ma) by the drug-ADE reference standard matrix (Mb) is retained in each cell the maximum value of the array-multiplication and the new matrix (Mc) containing the drug-ADE scores is obtained. As an example, “drug (a)” that causes the “ADE 2” is retrieved with a high score of 0.9. At the same time, “drug (a)” associated with the “ADE 3” (new candidate not present in the initial reference standard) is scored also with 0.9.

A multi-type drug-ADE predictor was generated through the integration of the previous data (see Figure 2a). We extracted the maximum value in each cell-summation-array calculated through the multiplication of the matrices Ma (3D similarity matrix) by Mb (ADE reference standard matrix). The new matrix called Mc contains in each cell the predictive score by each drug-ADE pair. We only retained the maximum value in each cell-array to generate only one leave-one-out score for each drug-ADE pair, provided by the most similar drug that causes the ADE. However, the method allows the implementation of alternative algorithms. Figure 2b shows how the integration of the data generated a final multi drug-ADE predictor. For instance, drug (a) that causes the ADE 1 in the example generates as a candidate the pair “ADE 1-drug (c)” with a score of 0.9, which is the 3D similarity score between drug (a) and (c).

### Application of the multi-ADE predictor in pharmacovigilance data

Offsides database^27^ was used as the module that provided pharmacovigilance data, an off-label adverse effects resource with 1,332 drugs and 10,097 adverse effects. This database was generated previously through mining FAERS^18^. Offsides provides a set of drug-ADE cases with the respective Empirical Bayes Geometric Mean (EBGM), *t*-statistics, and *p*-values. Our 3D database extracted from Mc matrix was mapped to the pharmacovigilance data extracted from Offsides, retaining 53,692 drug-ADE associations in common.

### Evaluation of the performance

We calculated the area under the receiver operating characteristic (AUROC) curve as a comparative measure of the quality of the performance in different situations: 1) for the 3D multi-ADE predictor considering all the generated drug-ADE scorings as an outcome, 2) for the 3D multi-ADE predictor considering each ADE as an individual outcome 3) for the global set of drug-ADE associations in common between Offsides and the 3D predictor as an outcome, and 4) for the drug-ADE associations in common between both data sources but considering each ADE as an individual outcome for plotting the results. Box plots were created to visualize the AUROC results for the individual ADEs. When the 3D model was applied to Offsides, the drug-ADE associations were ranked according to Empirical Bayes Geometric Mean (EBGM), *t*-statistic, *p*-values and the 3D score. Precision was also calculated in different top positions to evaluate the quality of the results.

### Comparative modeling

*2D MACCS models:* ADE models were also generated integrating a 2D similarity matrix into the ADE reference standard data as explained previously. For the generation of the 2D similarity matrix, we calculated for each drug the molecular fingerprint MACCS^28^. The fingerprint represents each molecule as a bit vector that codifies the presence or non-presence of structural keys (values 1 and 0 respectively). The Tanimoto Coefficient (TC) was calculated to compare drug fingerprints and generate the 2D similarity matrix. More details about 2D MACCS similarity can be found in previous publications^16^.

*Quantitative structure-activity relationship (QSAR) models:* QSAR models for 10 ADEs were developed. Statistical analyses were performed with the help of SPSS [IBM SPSS Statistics for Windows, Armonk, NY: IBM Corp] and R packages [The R Project for Statistical Computing: http://www.r-project.org]. The database was divided in training (80% of the data) and test set (20%). We calculated with E-Dragon [E-Dragon Software, Virtual Computational Chemistry Laboratory: http://www.vcclab.org/lab/edragon] different types of molecular descriptors, i.e. including topological and physico-chemical descriptors. In the Linear Discriminant Analysis (LDA) we used a stepwise method for variable selection. Quality of the obtained functions was assessed with Fisher ratio (F), Wilks' statistic (U), level of significance (*p*-values) and ROC curves. A maximum of five independent variables (molecular descriptors) were introduced in the models. A more detailed explanation of the molecular descriptors can be found in the literature^29^. The parameters extracted from the QSAR models are: allergic contact dermatitis (U = 0.78, F (5,659) = 38.10, *p* < 0.001, descriptors: F-081, nRCO, O-061, nArNO2, nCrq), extrapyramidal symptoms (U = 0.82, F (5,659) = 28.75, *p* < 0.001, descriptors: nRNR2, nR07, D/Dr07, nN = C-N<, C-026), hyperpyrexia (U = 0.91, F (5,659) = 13.49, *p* < 0.001, descriptors: MAXDP, nRNR2, MATS1v, GATS1e, MATS3p), hypertrichosis (U = 0.80, F (5,659) = 32.59, *p* < 0.001, descriptors: nRCO, F-081, C-012, ww, GATS8e), increased intraocular pressure (U = 0.88, F (5,659) = 17.24, *p* < 0.001, descriptors: nRCO, EEig15d, nOHp, nCrq, GATS7p), malignant syndrome (U = 0.88, F (5,659) = 18.01, *p* < 0.001, descriptors: nRNR2, T(O..Br), GATS5e, HNar, MPC09), mydriasis (U = 0.88, F (5,659) = 17.44, *p* < 0.001, descriptors: Ms, EEig14d, H-047, nN, nR07), paralytic ileus (U = 0.73, F (5,659) = 49.94, *p* < 0.001, descriptors: nR11, nRNR2, Wap, MAXDP, H-053), pseudotumor cerebri (U = 0.80, F (5,659) = 33.85, *p* < 0.001, descriptors: nRCO, nOHt, BEHm2, H-051, nCconj), tardive dyskinesia (U = 0.87, F (5,659) = 18.90, *p* < 0.001, descriptors: nRNR2, nN = C-N<, F-084, MAXDP, nIsoxazoles).

## Results

### Performance of the multi-ADE predictor

**Figure 3:**
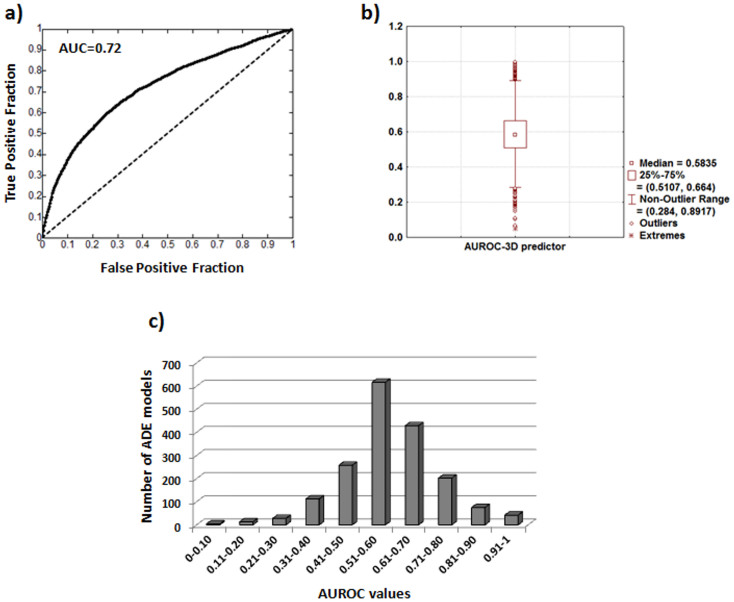
Panel a) Area under ROC curve for the 3D multi-ADE model; Panel b) Box plot of the AUROCs calculated for each individual ADE. Panel c) Graphic representing number of individual ADE models in each range of AUROC values.

We implemented the 3D drug similarity data in the initial ADE reference standard extracted from SIDER as described in the Methods. The model generates a new 853×1,780 drug-ADE matrix with 1,518,340 leave-one-out scores. Out of the total number of drug-ADE pairs, we labeled as true positives the initial SIDER drug-ADEs and as false positives the rest of possible drug-ADE pairs. We showed the area under the ROC curve (AUROC = 0.72) in Figure 3a. A validation was carried out through a hold-out method. We randomly introduced the 80% of the data in the model whereas the 20% was extracted to a test set. The AUROCs for the training and test sets were 0.71 and 0.73 respectively, showing a high degree of stability and agreement with the leave-one-out method.

Moreover, we also evaluated the performance of the model in each individual ADE. For each ADE we calculated the AUROC and showed the results in a box plot (see Figure 3b) with the median (0.58), the first quartile (0.51) and the third quartile (0.66). The number of ADE models in each range of AUROC values is represented in Figure 3c. Out of the 1,780 individual ADE models, 748 and 198 models presented an AUROC greater than 0.6 and 0.75 respectively.

**Figure 4:**
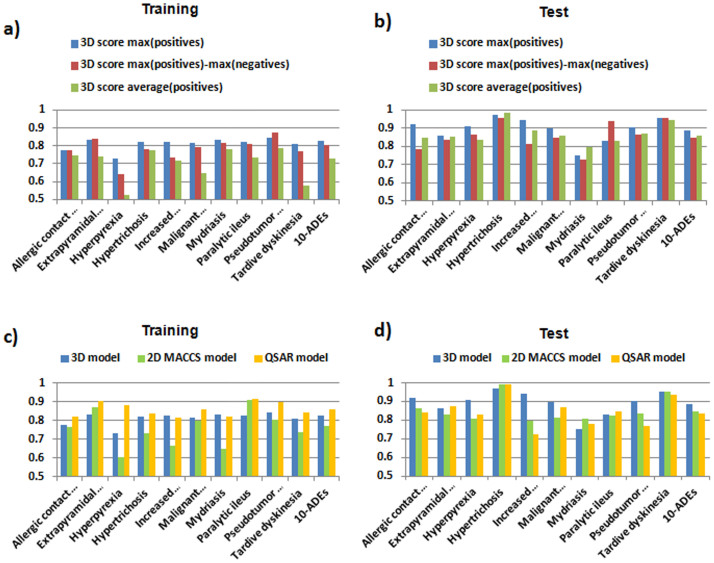
Panels (4a) and (4b): AUROC values in training and test sets using different algorithms in the 10 selected ADEs along with a global model including the 10 endpoints (algorithms: maximum similarity against the positive controls, difference between the maximum similarity against positive and negative controls, and average similarity against the positive controls). Panels (4c) and (4d): AUROC results in training and test sets for the 3D, 2D MACCS and QSAR modeling in the selected ADEs along with a global model including the 10 endpoints. The 10 selected ADEs are: allergic contact dermatitis, extrapyramidal symptoms, hyperpyrexia, hypertrichosis, increased intraocular pressure, malignant syndrome, mydriasis, paralytic ileus, pseudotumor cerebri and tardive dyskinesia.

Besides the maximum 3D score used in our models, we evaluated the performance of alternative 3D algorithms, including a double similarity score (defined as the difference between the maximum similarity against the positive and negative groups) and an average score (defined as the average similarity calculated against the positive group). Alternative algorithms were tested for the top 10 models with at least 25 drugs in the positive group. The data are divided into a training set (80%) and a test set (20%). Figures 4a and 4b show the AUROC values for the three algorithms in the 10 selected ADEs along with a global model including the 10 endpoints. The double similarity score performs similarly to the 3D maximum score, whereas the average score yields more limited results (see Figure 4a). A score including positive and negative controls was previously used by our research group with good results^24^. However, in our reference standard provided by SIDER database, the non-ADE drugs are really unknown. This fact could be a limitation factor for the use of alternative scores taking into account negative controls. For this reason, our 3D models are based on the maximum similarity against the set of drugs that cause the ADE with the aim of prioritizing drugs similar to the ADE drugs.

We also compared the performance using other types of similarities between drugs in the development of ADE predictors. As an example, AUROC global results for the 3D model were compared against a model developed using 2D molecular structure similarity, calculated through MACCS molecular fingerprints^28^. The global AUROC was 0.77, slightly better than the area determined with the 3D structure (AUROC = 0.72). In a similar way as described previously, we developed QSAR models for the top 10 3D endpoints with at least 25 drugs responsible for the ADE. Molecular descriptors were calculated with E-Dragon and Linear Discriminant Analysis (LDA) was used to generate 10 classifier functions. We calculated AUROC curves using the posterior classification probabilities extracted from the LDA. Figures 4c and 4d show the AUROC results for the 3D, 2D MACCS and QSAR modeling for the 10 different ADEs along with a global model including the 10 endpoints. The database in each ADE is divided in two sets: training (80%) and test set (20%). The different methods yielded similar results for the selected models. The average AUROCs for the 10 ADEs are 0.81, 0.75 and 0.86 in the training with the 3D, 2D and QSAR models respectively. In the test set, the average AUROC values are 0.89, 0.85 and 0.84 using 3D, 2D and QSAR modeling.

**Figure 5:**
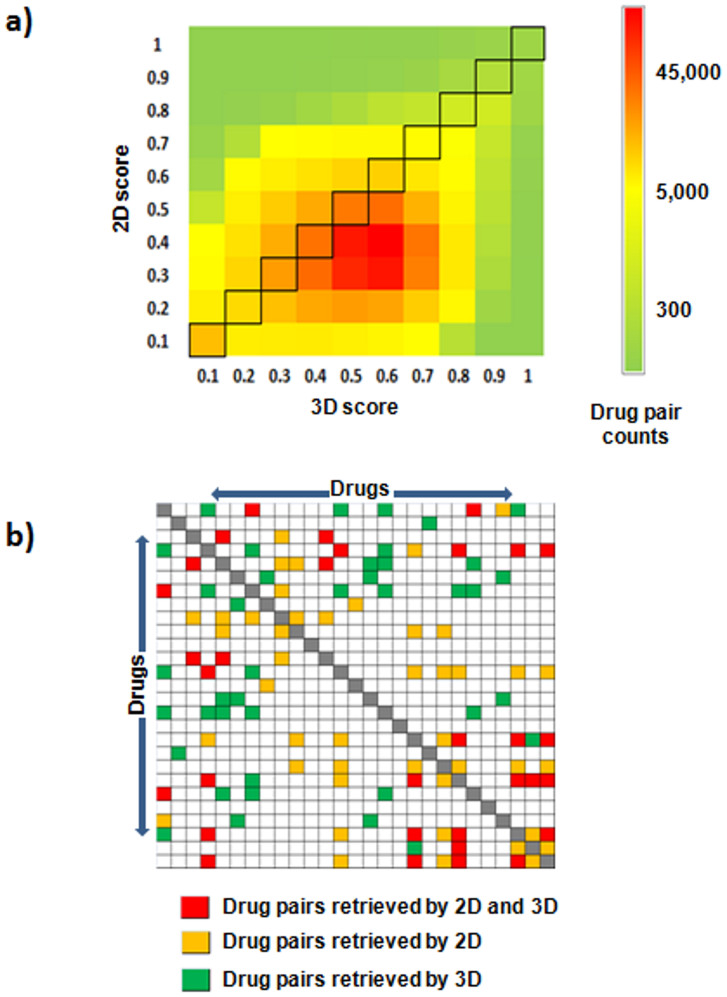
Comparison between the similarities obtained using 2D MACCS and 3D methods for all the pairs of drugs included in the study (panel a). Panel b shows the overlap in the top 10% similarities detected by the 3D and 2D approaches (MACCS) for the set of drugs responsible for the ADE hyperpyrexia. Drug pairs detected by both methods are colored in red. In orange is represented the pairs pointed out only by the 2D method. Pairs of drugs detected by the 3D method in the top 10% are colored in green. The matrix is symmetric and the diagonal is represented in grey.

The different perspective provided by the analysis of the 3D drug similarity can generate different sets of potential drug-ADE candidates. Figure 5a shows a comparison between the molecular similarities for all the pairs of drugs included in the study calculated using 2D MACCS and 3D methods. As shown in Figure 5a, some similar pairs are found with 3D methods (the 3D score is 0.7–0.8) and not detected with high 2D score (the score is 0.3–0.4), and vice versa. We also compared in Figure 5b the overlap in the 10% top similarities detected by the 3D method against the 2D approaches (MACCS fingerprint) for the set of drugs responsible for the ADE hyperpyrexia. Although both methods detected some pairs of drugs in common, there are differences in the detection of drug-drug similarities.

### Application of the multi-ADE predictor in Offsides database

We analyzed 53,692 drug-ADE pairs present in common in the 3D multi-ADE predictor and Offsides database^27^ (560 ADEs and 639 drugs in common), a resource of drug-ADE associations extracted from mining FAERS. As previously, the drug-ADE pairs present in SIDER were labeled as TP (7,714 cases) and the pairs not described in SIDER were deemed as FP (45,978 cases). The prevalence in the set of 53,692 drug-ADE associations is 0.14. This selected set is an enriched set of drug-ADE associations compared to the initial reference standard (matrix Mb) in which the prevalence was 0.057 (enrichment factor = 2.5).

**Figure 6:**
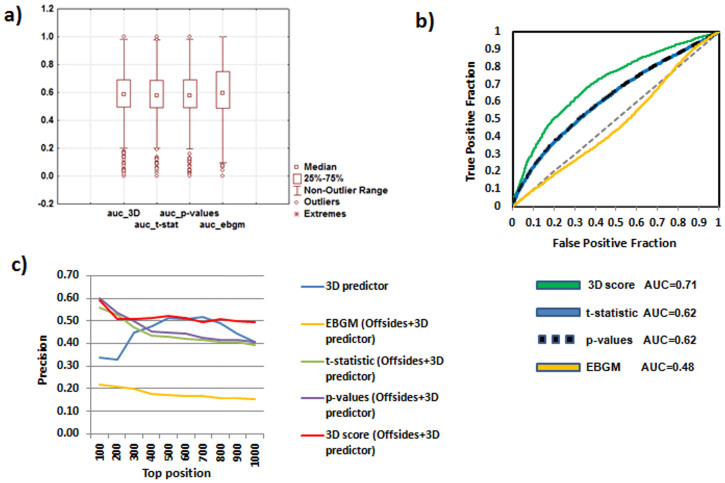
Data extracted from the drug-ADE associations found in both 3D multi-ADE model and Offsides database. Panel a) Box plot of the AUROCs calculated for each individual ADE using different methods to rank the drug-ADE associations: Empirical Bayes Geometric Mean (EBGM), *p*-values, *t*-statistic and our 3D similarity score. Panel b) Comparison of the ROC curves ranking the drug-ADE associations using Empirical Bayes Geometric Mean (EBGM), *p*-values, *t*-statistic and the 3D similarity score. Panel c) Precision achieved for the different methods in top positions 100–1000 (methods: EBGM, *p*-values, *t*-statistic and 3D score were applied to the set 3D multi-ADE model + Offsides. The precision using only the 3D multi-ADE predictor without Offsides was also plotted for comparative results). The method that offered the best precision results was 3D score in 3D multi-ADE model + Offsides.

Drug-ADEs were ranked using the Empirical Bayes Geometric Mean (EBGM), *t*-statistic and *p*-values extracted from Offsides and using the 3D score provided by our multi-ADE model previously generated. Although the AUROCs calculated for the individual ADEs showed similar results between EBGM, *t*-statistic, *p*-values and 3D scores (see Figure 6a with the box plot for all the methods), the best ranking plotting all the drug-ADE candidates in a global ROC curve was achieved with the 3D score (Figure 6b). The 3D score provided by our model showed an AUROC of 0.71, whereas the global AUROCs for *t*-statistic and *p*-values were 0.62 in both cases. Remarkably, the EBGM algorithm showed a poor performance with an AUROC = 0.48. As we have shown above, the EBGM used in Offsides was a good measurement to extract an enriched set of drug-ADE candidates, but it does not provide in this analysis the best approach to rank the drug-ADE associations.

Moreover, the precision in different top positions when all the drug-ADEs are merged was also analyzed as a comparative measurement (see Table 1 and Figure 6c). The results indicate that ranking the Offsides candidates with the 3D score is useful to obtain a more enriched subset of drug-ADE candidates.

## Discussion

In this paper, we showed a simple and efficient method to develop a 3D multi-ADE predictor with possible applications in pre-clinical ADE screenings of experimental drugs, and patient safety through the evaluation of marketed drugs as ADE candidates extracted from pharmacovigilance data sources. Although the 3D multi-type predictor was applied to a set of ADE candidates from mining FAERS (Offside data), other types of pharmacovigilance resources can be used, such as Electronic Health Records^17^. The application of our 3D multi-ADE predictor to Offsides resource allowed us to obtain enriched sets of ADE candidates interesting for further study. Moreover, since the 3D score provided by our models is based on the maximum similarity against the set of drugs that cause the ADE, we can isolate the drug that produced the signaling score and analyze if the ADE information available for it could be applicable to our drug candidate, pointing out possible ADE mechanisms of action. However, application of other considerations useful to evaluate the importance of the ADE candidate, such as pharmacological relationships or the study of the candidate in different data resources, is advisable for a more clear establishment of a “red flag” in the ADE candidate.

Performance of the 3D multi-ADE predictor is highly dependent on the quality of the initial ADE reference standard used to develop the model. Different complexity in each ADE of the reference standard, related to the molecular structure and pharmacological categories of the drugs collected for each ADE, is responsible for an irregular performance since the different ADEs showed AUROCs from poor to excellent values. With the integration of 3D pharmacophoric similarity in the SIDER database, we maximize the pattern “similar drugs have similar ADEs” already present in some of the studied ADEs, but not in all of them; hence, the variable performance of the 3D predictor in the individual ADEs. Moreover, further studies would be necessary to confirm some drug reactions in our reference standard provided by SIDER database, and the non-ADE drugs (negative control) are really unknown. Although SIDER is a very useful publically available resource, missing data problem is a limitation inherent to the predictive models developed with the reference data. Prevalence in SIDER is variable depending on the ADE and spans values between 0.001 for some reactions, such as abasia, and 0.82 for the ADE nausea. Additional enhancement in the representativeness of the initial reference standard database could improve the external predictive power in this type of predictor.

Other types of similarities between drugs can be included to develop ADE models. Pauwels et al.^15^ developed a multi-ADE predictor integrating the SIDER database with 2D molecular similarity information extracted from 2D fingerprints defined in PubChem^30^. They integrated the information through the use of different methods, such as nearest neighbor, support vector machines and canonical correlation analysis. 2D methods showed excellent global ROC curves and the method showed potential to generate correlated sets of chemical fragments and adverse effects.

Although the use of 2D molecular structure has been proven to offer good results avoiding complex calculations, the 3D molecular structure analysis provides different insights regarding to 2D analysis. As shown in Figure 5, both methods, 2D and 3D, are able to detect a diverse chemical space. Some studies also demonstrated relationships between chemical structures and biological functions that are not captured with 2D approaches but that become more detectable using the 3D molecular structure similarity^31^,^32^,^33^. The different perspective provided by the analysis of the 3D drug similarity can generate different sets of potential drug-ADE candidates.

**Figure 7:**
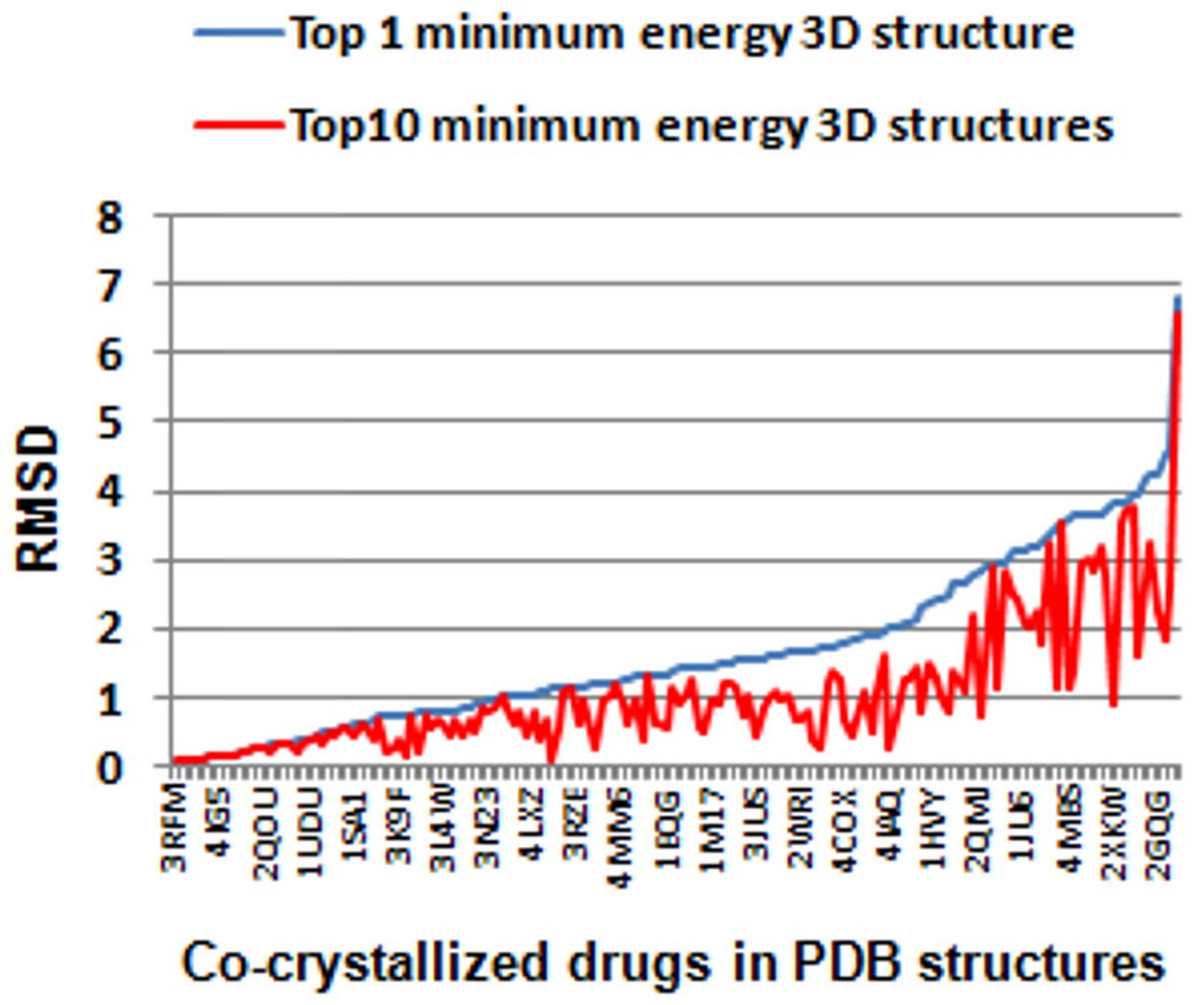
RMSD between co-crystallized drugs bound to protein targets and 1) the global minimum energy structure extracted from the MCMM conformational analysis (blue color, values sorted in increasing order), and 2) the top 10 minimum energy structures obtained in the MCMM calculation (red color). The minimum RMSD out of the 10 structures is plotted. 158 drugs in PDB structures were investigated and represented in the X axis.

For the development of the 3D predictor it is possible the use of alternative approaches in regards to the methods described in the current article, i.e. other types of conformational search engines, different algorithms for molecular alignment or the applicability of alternative similarity scoring functions. In this study, for simplicity, only the global minimum energy structure generated by the conformational analysis was retained as representative of the calculation. However, it is worth to point out that in many occasions the bioactive conformations of the molecules binding the protein are not captured with the simple retention of the global minimum energy conformation^34^. In some cases, it could be more convenient to retain more conformations extracted from the MCMM to detect with more precision the bioactive bound form of the drug. With the aim of performing a comparative study, we collected from the Protein Data Bank^35^ the 3D co-crystallized structure of 158 drugs included in our data bound to protein targets. We superimposed and measured the root mean square deviation (RMSD) between the global minimum energy 3D structures generated through the MCMM conformational analysis and the co-crystallized drug structures (see Figure 7). A comparative study was carried out taking into account the top 10 3D minimum energy conformations generated by the MCMM. Out of the 10 conformations, the best RMSD against the crystallized drugs is plotted in Figure 7. The average RMSD using the first and the second protocol are 1.66 and 1.05 respectively. The second protocol offered better RMSD results achieving drug conformations more in accordance with the co-crystallized bioactive conformations. However, it is still a challenge to predict which of the ten conformations is the closest to the crystallized drug conformation, increasing the complexity in posterior calculations. Moreover, the protocol followed in this study, considering only the minimum energy structure, is simpler and presented acceptable RMSD values compared to co-crystallized structures (112 out of 158 structures with a RMSD lower than 2). It is also worth noting than drugs could present different bioactive conformations depending on the receptor they are bound to. In fact, many ADEs are due to the interaction between drugs and off-targets with possible different drug conformational states depending on the target. Although docking simulations could be a method to calculate the 3D drug conformations, off-targets responsible for the ADEs are in many cases unknown due to the lack of information in ADE mechanisms of action.

In this article, we developed a 3D multi-ADE predictor based on the integration of 3D pharmacophoric similarity into an ADE reference standard provided by SIDER database. Combination of the 3D predictor with pharmacovigilance data allowed a better prioritization of multiple ADE candidates and the generation of enriched sets of ADE signals. These types of predictors are promising tools to analyze data from pharmacovigilance studies and rationalize the results. The method is simple and efficient, applicable to ADE large scale detection, and can help in the ADE decision making process to select some candidates for further studies.

## Author Contributions

S.V. and G.H. wrote the manuscript. S.V., N.P.T. and G.H. designed the research. S.V. performed the research. S.V. analyzed the data. S.V., N.P.T. and G.H. contributed new reagents/analytical tools.

## Figures and Tables

**Figure 1:**
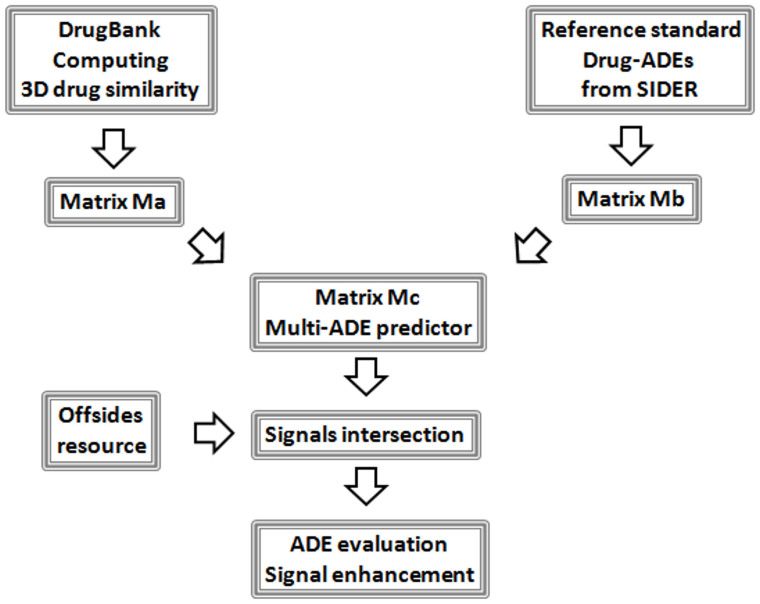
Workflow followed in the development of the current study.

**Table 1:** Precision of the different methods in top scoring positions

Precision (TP/TP + FP)					
TOP position	3D score^a^ (3D predictor by itself)	EBGM^b^ (Offsides + 3D predictor)	*t*-statistic^b^ (Offsides + 3D predictor)	*p*-values^b^ (Offsides + 3D predictor)	3D score^b^ (Offsides + 3D predictor)
100	0.34	0.22	0.56	0.60	0.59
200	0.33	0.21	0.53	0.54	0.51
300	0.45	0.20	0.47	0.50	0.51
400	0.48	0.18	0.44	0.45	0.51
500	0.51	0.17	0.43	0.45	0.52
600	0.51	0.17	0.42	0.45	0.51
700	0.52	0.17	0.42	0.43	0.50
800	0.49	0.16	0.41	0.42	0.51
900	0.44	0.16	0.41	0.41	0.50
1000	0.41	0.16	0.39	0.41	0.49

^a^Candidates generated by the 3D multi-ADE predictor without Offsides ranked according to the 3D score;

^b^candidates in common in the 3D multi-ADE predictor and Offsides ranked using EBGM, *t*-statistic, *p*-values and 3D score.

## References

[b1] Bates D. W. *et al.* The costs of adverse drug events in hospitalized patients. JAMA 277, 307–311 (1997). 9002493

[b2] Wu C., Bell C. M. & Wodchis W. P. Incidence and Economic Burden of Adverse Drug Reactions among Elderly Patients in Ontario Emergency Departments A Retrospective Study. Drug Saf. 35, 769–781 (2012). 22823502 10.1007/BF03261973 PMC3714138

[b3] Bass A. S. *et al.* Exploratory drug safety: A discovery strategy to reduce attrition in development. J. Pharmacol. Toxicol. Methods 60, 69–78 (2009). 19422924 10.1016/j.vascn.2009.04.194

[b4] Kuhn M. *et al.* Systematic identification of proteins that elicit drug side effects. Mol. Syst. Biol. 9, 663 (2013). 23632385 10.1038/msb.2013.10 PMC3693830

[b5] Wallach I., Jaitly N. & Lilien R. A. Structure-Based Approach for Mapping Adverse Drug Reactions to the Perturbation of Underlying Biological Pathways. PLOS ONE 5, e12063 (2010). 20808786 10.1371/journal.pone.0012063 PMC2925884

[b6] Lounkine E. *et al.* Large-scale prediction and testing of drug activity on side-effect targets. Nature 486, 361–7 (2012). 22722194 10.1038/nature11159 PMC3383642

[b7] Valerio L. G. Jr In silico toxicology for the pharmaceutical sciences. Toxicol. Appl. Pharmacol. 241, 356–370 (2009). 19716836 10.1016/j.taap.2009.08.022

[b8] Benigni R. & Bossa C. Predictivity and reliability of QSAR models: The case of mutagens and carcinogens. Toxicol. Mech. Methods 18, 137–147 (2008). 20020910 10.1080/15376510701857056

[b9] Jensen G. E., Niemela J. R., Wedebye E. B. & Nikolov N. G. QSAR models for reproductive toxicity and endocrine disruption in regulatory use - a preliminary investigation. SAR QSAR Environ. Res. 19, 631–641 (2008). 19061080 10.1080/10629360802550473 PMC2607135

[b10] Segall M. D., Beresford A. P., Gola J. M. R., Hawksley D. & Tarbit M. H. Focus on success: using a probabilistic approach to achieve an optimal balance of compound properties in drug discovery. Expert Opin. Drug Metab. Toxicol. 2, 325–337 (2006). 16866617 10.1517/17425255.2.2.325

[b11] Vilar S., Chakrabarti M. & Costanzi S. Prediction of passive blood-brain partitioning: Straightforward and effective classification models based on in silico derived physicochemical descriptors. J. Mol. Graph. Model. 28, 899–903 (2010). 20427217 10.1016/j.jmgm.2010.03.010 PMC2873098

[b12] Valerio L. G. Jr In silico toxicology models and databases as FDA Critical Path Initiative toolkits. Hum. Genomics 5, 200–207 (2011). 21504870 10.1186/1479-7364-5-3-200 PMC3500173

[b13] Scheiber J. *et al.* Mapping Adverse Drug Reactions in Chemical Space. J. Med. Chem. 52, 3103–3107 (2009). 19378990 10.1021/jm801546k

[b14] Bender A. *et al.* Analysis of pharmacology data and the prediction of adverse drug reactions and off-target effects from chemical structure. ChemMedChem 2, 861–873 (2007). 17477341 10.1002/cmdc.200700026

[b15] Pauwels E., Stoven V. & Yamanishi Y. Predicting drug side-effect profiles: a chemical fragment-based approach. BMC Bioinformatics 12, 169 (2011). 21586169 10.1186/1471-2105-12-169 PMC3125260

[b16] Vilar S. *et al.* Facilitating adverse drug event detection in pharmacovigilance databases using molecular structure similarity: application to rhabdomyolysis. J. Am. Med. Inform. Assoc. 18, I73–I80 (2011). 21946238 10.1136/amiajnl-2011-000417 PMC3241177

[b17] Vilar S., Harpaz R., Santana L., Uriarte E. & Friedman C. Enhancing Adverse Drug Event Detection in Electronic Health Records Using Molecular Structure Similarity: Application to Pancreatitis. PLOS ONE 7, e41471 (2012). 22911794 10.1371/journal.pone.0041471 PMC3404072

[b18] FDA U. S. Food and Drug Administration. FDA Adverse Event Reporting System (FAERS). Available at: http://www.fda.gov/cder/aers/default.htm (Accessed: Jun 2013).

[b19] Harpaz R. *et al.* Performance of Pharmacovigilance Signal-Detection Algorithms for the FDA Adverse Event Reporting System. Clin. Pharmacol. Ther. 93, 539–546 (2013). 23571771 10.1038/clpt.2013.24 PMC3857139

[b20] Hauben M. & Bate A. Decision support methods for the detection of adverse events in post-marketing data. Drug Discov. Today 14, 343–357 (2009). 19187799 10.1016/j.drudis.2008.12.012

[b21] Harpaz R. *et al.* Combing signals from spontaneous reports and electronic health records for detection of adverse drug reactions. J. Am. Med. Inform. Assoc. 20, 413–419 (2013). 23118093 10.1136/amiajnl-2012-000930 PMC3628045

[b22] Ryan P. B., Madigan D., Stang P. E., Schuemie M. J. & Hripcsak G. Medication-Wide Association Studies. CPT: Pharmacometrics Syst. Pharmacol. 2, e76 (2013). 24448022 10.1038/psp.2013.52 PMC4026636

[b23] Almenoff J. S., LaCroix K. K., Yuen N. A., Fram D. & DuMouchel W. Comparative performance of two quantitative safety signalling methods - Implications for use in a pharmacovigilance department. Drug Saf. 29, 875–887 (2006). 16970511 10.2165/00002018-200629100-00005

[b24] Vilar S. *et al.* Similarity-based modeling applied to signal detection in pharmacovigilance. CPT Pharmacometrics Syst. Pharmacol. 3, e137 (2014). 25250527 10.1038/psp.2014.35 PMC4211266

[b25] SIDER Side Effect Resource. Available at: http://sideeffects.embl.de/ (Accessed: May 2013).

[b26] DrugBank database, version 3.0. Available at: http://www.drugbank.ca/ (Accessed: Jul 2013).

[b27] Tatonetti N. P., Ye P. P., Daneshjou R. & Altman R. B. Data-Driven Prediction of Drug Effects and Interactions. Sci. Transl. Med. 4, 125ra31 (2012). 10.1126/scitranslmed.3003377 PMC3382018 22422992

[b28] Durant J. L., Leland B. A., Henry D. R. & Nourse J. G. Reoptimization of MDL keys for use in drug discovery. J. Chem. Inf. Comput. Sci. 42, 1273–1280 (2002). 12444722 10.1021/ci010132r

[b29] Todeschini R. & Consonni V. Handbook of Molecular Descriptors. Wiley-VCH, Weinheim, (Germany), 2000.

[b30] The PubChem Project. Available at: http://pubchem.ncbi.nlm.nih.gov/ (Accessed: Jul 2014).

[b31] Bolton E. E. *et al.* PubChem3D: a new resource for scientists. J. Cheminform. 3, 32 (2011). 21933373 10.1186/1758-2946-3-32 PMC3269824

[b32] Kim S., Bolton E. E. & Bryant S. H. PubChem3D: Biologically relevant 3-D similarity. J. Cheminform. 3, 26 (2011). 21781288 10.1186/1758-2946-3-26 PMC3223603

[b33] Vilar S., Uriarte E., Santana L., Friedman C. & Tatonetti N. P. State of the Art and Development of a Drug-Drug Interaction Large Scale Predictor Based on 3D Pharmacophoric Similarity. Curr. Drug Metab. 15, 490–501 (2014). 25431152 10.2174/138920021505141126102223

[b34] Vieth M., Hirst J. D. & Brooks C. L. Do active site conformations of small ligands correspond to low free-energy solution structures? J. Comput. Aided Mol. Des. 12, 563–572 (1998). 9879504 10.1023/a:1008055202136

[b35] RCSB Protein Data Bank. Available at: http://www.rcsb.org/ (Accessed: Dec 2014).

